# The Impact of Bevacizumab (Avastin) on Survival in Metastatic Solid Tumors - A Meta-Analysis and Systematic Review

**DOI:** 10.1371/journal.pone.0051780

**Published:** 2013-01-22

**Authors:** Limor Amit, Irit Ben-Aharon, Liat Vidal, Leonard Leibovici, Salomon Stemmer

**Affiliations:** 1 Institute of Oncology, Davidoff Center, Rabin Medical Center, Beilinson Campus, Petach Tiqwa, Israel and the Sackler School of Medicine, Tel–Aviv University, Israel; 2 Institute of Hematology, Davidoff Center, Rabin Medical Center, Beilinson Campus, Petach Tiqwa Israel and the Sackler School of Medicine, Tel–Aviv University, Israel; 3 Medicine E Rabin Medical Center, Beilinson Campus, Petach Tiqwa, Israel and the Sackler School of Medicine, Tel–Aviv University, Israel; University Clinic of Navarra, Spain

## Abstract

**Purpose:**

To evaluate the effect of Bevacizumab in combination with chemotherapy on overall survival of patients with metastatic solid tumors.

**Design:**

A systematic literature search to identify randomized trials comparing chemotherapy with and without Bevacizumab in metastatic cancer. The primary end point was overall survival (OS) and the secondary end points were progression free survival (PFS) and toxicity. A meta-analysis was performed for each tumor type and for the combination of all tumors.

**Results:**

24 randomized trials with 8 different types of malignancies were included in this meta-analysis. Patients treated with Bevacizumab had an OS benefit, hazard ratio (HR) 0.89 (95% CI 0.84–0.93, P<0.00001 I^2^-4%). The combined analysis showed a PFS benefit with a HR 0.71 (95% CI 0.68–0.74, P<0.00001, I^2^-54%). The toxicity analysis showed a statistically significant increase in fatal adverse events (FAEs) in the Bevacizumab treatment arm, risk ratio (RR) 1.47 (95% CI 1.1–1.98). A separate analysis of the lung cancer trials showed an increased risk of fatal pulmonary hemorrhage with a RR of 5.65 (95% CI 1.26–25.26). The risk of G3–4 adverse events was increased: RR 1.2 (95% CI 1.15–1.24).

**Conclusion:**

in this combined analysis Bevacizumab improved OS (with little heterogeneity) and PFS. These results should be considered in the light of lack of markers predictive of response and the increased severe and fatal toxicity seen with Bevacizumab treatment.

## Introduction

Neovascularization is one of the main mechanisms for the progression of human solid tumors and also provides a pathway for the migration of tumor cells by accessing the systemic circulation to establish distant metastases. Vascular endothelial growth factor (VEGF) plays an essential role in angiogenesis [1]–[5]. Bevacizumab is a humanized monoclonal antibody that blocks the binding of VEGF to its receptors and results in regression of immature tumor vasculature, normalization of remaining tumor vasculature and inhibition of further tumor angiogenesis [6]. The complete mechanism of angiogenesis inhibition is not entirely understood. Due to the proposed universal anti-tumor activity of Bevacizumab it was widely studied in the treatment of early and metastatic tumors.

Several randomized controlled trials have evaluated the role of Bevacizumab in addition to chemotherapy for patients with metastatic colorectal cancer [7]–[13]. A recent meta-analysis found a statistically significant median OS advantage for patients with metastatic colorectal cancer of 20.5 months with Bevacizumab compared with 17.7 months without - with a hazard ratio (HR) for overall survival (OS) of 0.81 and for progression free survival (PFS) of 0.6 [14].

The role of angiogenesis is established in the progression of lung cancers [15]. Four randomized controlled studies [16]–[20] evaluated the role of Bevacizumab in metastatic NSCLC yielding conflicting results in terms of survival benefit. The first study showed that squamous cell (SCC) histology had a high risk for fatal (mostly bleeding) events when treated with Bevacizumab. Therefore the following trials excluded patients with SCC. The ECOG 4599 study showed a survival advantage for Bevacizumab combined with Carboplatinum and Paclitaxel. The AVAIL study combined Bevacizumab with Cisplatinum and Gemcitabine (which is less effective in adenocarcima [21]) and showed a very small PFS advantage and no OS benefit. Following those studies the FDA approved the use of Bevacizumab in metastatic adenocarcinoma of lung.

In metastatic breast cancer patients, few randomized controlled trials appraised the use of Bevacizumab as first-line treatment in combination with chemotherapy agents. In general these studies showed improvement in tumor response rate and PFS but not OS [22]–. The combination of Taxanes or Capecitabine with Bevacizumab until progression seems to result in the best PFS in this setting. Another recent metaanalysis in metastatic breast cancer failed to show a significant benefit in OS [32]. Therefore the FDA has recently revoked the recommendation for the use of Bevacizumab in first line metastatic breast cancer.

Bevacizumab is an attractive option for metastatic renal cell carcinoma because of the correlation between VEGF and von Hippel Lindau (VHL) tumor suppressor gene, which has a substantial role in the mechanism of the disease. Two phase III trials were performed [33]–[37] evaluating the role of Bevacizumab in combination with INFα compared to INFα alone. These trials showed a PFS benefit but no OS advantage.

In pancreatic cancer, two phase III studies combining Gemcitabine with Bevacizumab showed negative results with no increase in OS [38]–[39].

VEGF expression is a negative prognostic factor for survival in patients with gastric cancer. A preliminary phase II trial showed encouraging results [40], but the phase III trial showed a significant ORR benefit (46% vs 37%, P = 0.0315) without survival benefit [41].

In metastatic castrate resistant prostate cancer (mCRPC) patients preclinical activity of VEGF blockade and inverse relationship of plasma and urine VEGF levels and survival suggested that VEGF blockade was an appropriate potential strategy. A recent phase III trial evaluated approximately 1000 patients and failed to show a significant OS benefit [42].

Malignant melanoma is a highly vascular tumor in which VEGF is expressed and seems to play a role in disease progression. BEAM - a randomized phase II study in patients with previously untreated metastatic melanoma compared Carboplatinum and Paclitaxel with and without Bevacizumab [43]. This trial did not reach its primary objective of statistically significant improvement in PFS.

Due to the non specific mechanism of action of Bevacizumab in solid tumors and the conflicting overall survival results, we aimed to perform a meta-analysis of all available data regarding the efficacy and toxicity of Bevacizumab in metastatic solid tumors. A significant effect without heterogeneity across all tumor types would increase the confidence in the efficiency of Bevacizumab.

## Methods

### Outcome measures

The primary outcomes of this meta-analysis were overall survival (OS), defined as time from randomization to death; and progression free survival (PFS) defined as the continued survival in the absence of evidence for progression of disease. Secondary outcomes were response rate (RR) – the percentage of patients achieving a complete response or a partial response defined by RECIST criteria (Response Evaluation Criteria in Solid Tumors) and toxicity. Toxicity was defined as grade >2 (hematological and non-hematological), including adverse events that may be typical to Bevacizumab (as hypertension and vascular events). The number of events was divided by number of patients analyzed.

### Literature search

Relevant randomized clinical trials were identified by searching The Cochrane Library (12/2011), MEDLINE (January 1966 to Dec 2011) and LILACS (12/2011). The terms ‘Bevacizumab OR Avastin’ and similar, and *‘cancer’* and similar were crossed (see appendix S1 for search strategy). A search for abstracts was performed in the proceedings of the following conferences: American society of clinical oncology (ASCO), European society of medical oncology (ESMO) and San Antonio Breast Cancer Symposium. National cancer institute was searched 12/2011 for any ongoing trials. References of selected articles were reviewed for any additional relevant trials, and original authors were contacted for possible unpublished data.

### Inclusion criteria

We included all randomized controlled trials that compared the addition of bevacizumab to various chemotherapy protocols without Bevacizumab in adult patients with metastatic cancer in the first and second line setting seperately. We excluded brain tumors due to the different drug delivery through the blood brain barrier and due to its role in reducing edema around the tumor [56]. We also excluded ovarian cancer as the trials included metastatic and non metastatic disease. We excluded maintenance therapy trials and phase II non randomized and phase IV trials. Trials in which the primary outcome measure of OS was not reported were included if all other inclusion criteria were met. Trials assessing toxicity alone were excluded.

### Data extraction

Two reviewers independently applied inclusion criteria, selected the studies, and extracted data and outcomes. In case of disagreement between the two reviewers, a third reviewer extracted the data. Authors of studies were contacted when clarification was needed or for providing complementary data.

### Quality assessment

Trials fulfilling the review inclusion criteria were assessed for methodological quality by two reviewers. For RCTs, data regarding randomization and allocation concealment, blinding, sample size, exclusions after randomization, and different lengths of follow-up were performed using the criteria described in the Cochrane Reviewer's handbook.

### Statistical methods

Hazard ratios (HR) and variances for time to event outcomes were estimated as described by Parmar et al, and pooled according to inverse of variance method (Review Manager [RevMan], version 5 for Windows; The Cochrane Collaboration, Oxford, United Kingdom). Pooled relative risk (RR) and 95% confidence intervals (CI) for dichotomous data were estimated using the Mantel-Haenszel method. The confidence intervals for OS from three studies were calculated from the published Kaplan Mayer curves [7], [12], [17]. We assessed heterogeneity in the results of trials by inspection of their graphical presentations and by calculating a Chi^2^ test of heterogeneity and the *I* ^2^ measure of inconsistency. Significant heterogeneity was defined as a Chi^2^ test *P*<.1 or an *I* ^2^ measure greater than 50%^17^. We used a fixed effect model to pool results except in the event of significant heterogeneity when random-effects model was used (inverse of variance method and DerSimonian and Laird method). Analysis was performed for all included studies and for each cancer type for each available outcome.

For calculating number needed to harm (NNH) in order to evaluate the additive effect of Bevacizumab on the absolute risk for grade 3–4 adverse events, we retrieved the data on the incidence data of grade 3–4 adverse events in each arm (Bevacizumab versus control). Risk was calculated by multiplying the absolute risk of the control arm by (1-RR for fatal adverse events).

## Results

**Figure 1:**
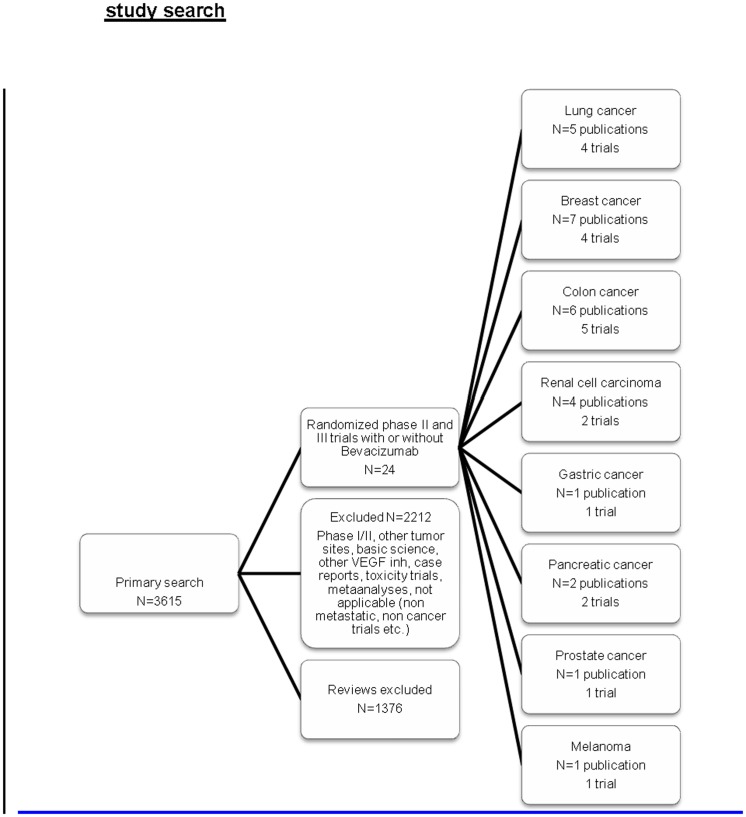
Trial selection.

Our primary search yielded 3615 publications of which 1376 were reviews. We excluded 2212 publications as not relevant, leaving 27 publications for 24 relevant studies to this analysis (figure 1). The included studies were randomized phase II or phase III studies of Bevacizumab in combination with chemotherapy in the metastatic setting. Study characteristics are presented in table 1. One study [9] was eventually excluded from the efficacy analysis but included in the toxicity analysis as survival data was lacking.

**Table 1:** Trials protocols & data summary.

Study	Phase	Tx line	# pts	Protocol	Bev duration	PFS (m) (net improve m) (% improve)	OS (m) (net improve m) (% improve)	Outcome
**NSCLC**								
**Johnson 2004 (19)**	II	1^st^	99	CP bev 7.5/15 mg/kg	6 cycles	TTP non SCC 4 m, 6.3 m, 7.1 m for HD p = 0.01 (+2.3/+3.1) (+57%/77.5%)	Non SCC 12.2 m, 14 m 17.8 m NS	Pos
**Sandler 2006 (18)**	III	1^st^	878	CP Bev 15 mg/kg	6 cycles	4.5 m vs 6.2 m p<0.001 (+37%) (+1.7)	10.3 m vs 12.5 m (+22%) p = 0.003 (+2.3)	Pos
**Herbst 2007 (20)**	II	2^nd^	122	D/Pa/E Bev 15 mg/kg	1 year	3/4.8/4.4 NS	8.6/12.6/13.7 NS	Pos
**Reck 2009 (16–17)**	III	1^st^	1043	CG Bev 7.5/15 mg/kg	6 cycles	6.1/6.7/6.5 p = 0.003 (+0.6/0.4) (9.8%/6.5%)	13.1/13.6/13.4 NS	Pos
**Breast cancer**								
**Miller 2005 (22)**	III	3^rd^	462	Cape/Bev 15 mg/kg	35 cycles	4.1/4.8NS	14.5/15.1 NS	Pos
**Miller 2007, Gray 2009 (23–24)**	III	1^st^	722	Pac/Bev 15 mg/kg	Till PD	5.9/11.8 p<0.001(+5.9) (+100%)	25.2/26.7 NS	Pos
**Miles 2010 (28)**	III	1^st^	736	D/Bev 7.5/15 mg/kg	9 cycles	8.2/9/10.1 p<0.05 (+0.8/1.9) (+9.7%/23%)	31.9/30.8/30.2 NS	Pos
**Robert 2009 (RIBBON 1) (25–27)**	III	1^st^	1237	Cape/anthra+Tax Bev 15 mg/kg	Till PD	5.7/8.68/9.2 p<0.05 (+2.9/1.2) (+50.8%/15%)	21/29 23.8/25.2 NS	Pos
**Brufsky 2010 (RIBBON 2) (29–31)**	III	2^nd^	684	Chemo +Bev 10 or 15 mg/kg	Till PD	5.1/7.2 p = 0.0072 (+2.1) (+41%)	17.8/18.6 NS	Neg
**Colon cancer**								
**Kabbinavar 2003 (9)**	II	1^st^	104	5FU/LCV/Bev 5/10 mg/kg	6 cycles	TTP 5.2/9/7.2 p = 0.05/0.217 (+3.8/2) (+73%/38%)	13.8/21.5/16.1 NS (+7.7/2.3) (+55.7%/14%)	Pos
**Kabbinavar 2005 (10–11)**	II	1^st^	209	5FU/LCV Bev 5 mg/kg	96w	5.5/9.2 p = 0.0002 (+4.7)(+85%)	12.9/16.6NS	Pos
**Hurwitz 2004 (7)**	III	1^st^	813	IFL Bev 5 mg/kg	96w	6.2/10.6 p<0.001 (+4.4)(+70%)	15.6/20.3 p<0.001 (+4.7)(+30%)	Pos
**Saltz 2008 (8)**	III	1^st^	1401	FOLFOX/XELOX Bev 5 mg/kg	56w	TTP 6/6.9 p = 0.003 (+0.9) (+15%)	19.9/21.3 NS	Pos
**Tebbutt 2010 (13)**	III	1^st^	313	Capecitabine/Bev 7.5 mg/kg (q3w)	Till PD	5.7/8.5 p<0.01 (+2.8) (+49%)	18.9/18.9	Pos
**Renal cell carcinoma**								
**Rini 2008/2010 (34–35)**	III	1^st^	732	INF Bev 10 mg/kg	Till PD	5.2/8.5 p = 0.001 (+3.3) (+63.4%)	17.4/18.3NS	Neg
**Escudier 2007/2010 (36–37)**	III	1^st^	647	INF Bev 10 mg/kg	52w	5.4/10.2 p = 0.0001 (+4.8)(+88%)	21.3/23.3NS	Pos
**Gastric cancer**								
**Atsushi 2011 (41)**	III	1^st^	774	XP Bev 7.5 mg/kg	Till PD	5.3/6.7 p = 0.0037 (+1.4) (+26.4%)	10.1/12.1NS	Neg
**Pancreatic cancer**								
**Kindler 2010 (39)**	III	1^st^	602	Gem Bev 10 mg/kg	Till PD	2.9/3.8NS	5.9/5.8NS	Neg
**Van-Cutsem 2009 (38)**	III	1^st^	301	Gem/E Bev 5 mg/kg	Till PD	3.6/4.6 p = 0.002 (+1)(+27.7%)	6/7.1 NS	Neg
**Prostate cancer**								
**Kelly 2010 (42)**	III	1^st^	1050	D prednisone Bev 15 mg/kg	Till PD	7.5/9.9 p<0.0001 (+2.4) (+32%)	21.5/22.6 p = 0.181 (+1.1) (+5.1%)	Neg
**Melanoma**								
**Kim 2011 (43)**	II	1^st^	214	CP Bev 15 mg/kg	Till PD	4.2/5.6 NS	9.2/12.3 NS	Neg

### Efficacy

OS analysis included 19 trials and 11,422 patients with metastatic solid tumors treated with chemotherapy alone or with Bevacizumab and yielded a significant effect in OS favoring Bevacizumab, HR 0.89 (95% CI 0.84–0.93 P<0.00001) with low heterogeneity I^2^ = 4%.

**Figure 2:**
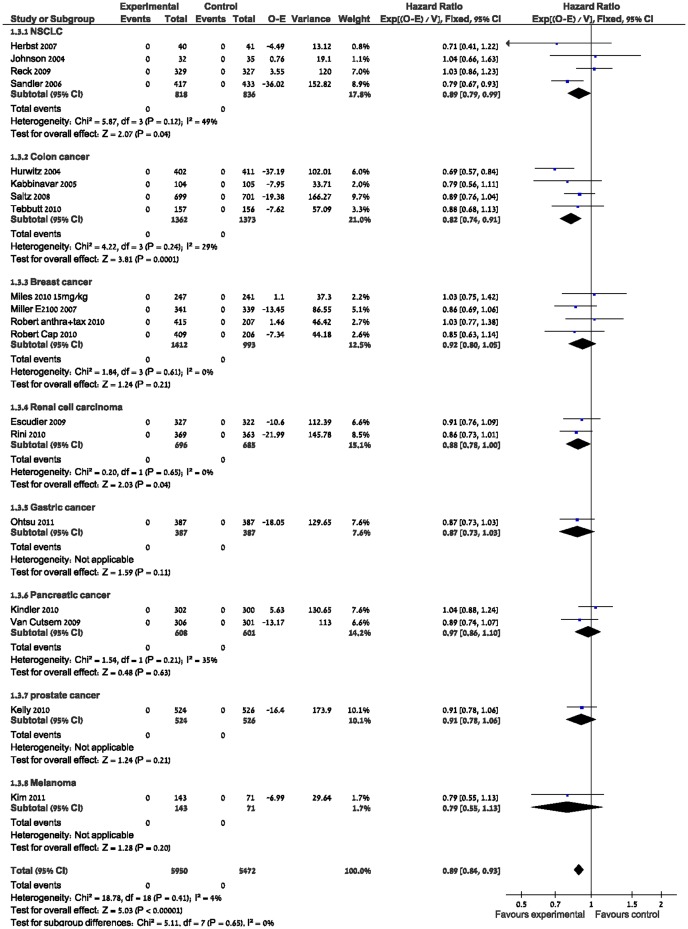
Overall survival.

**Figure 3:**
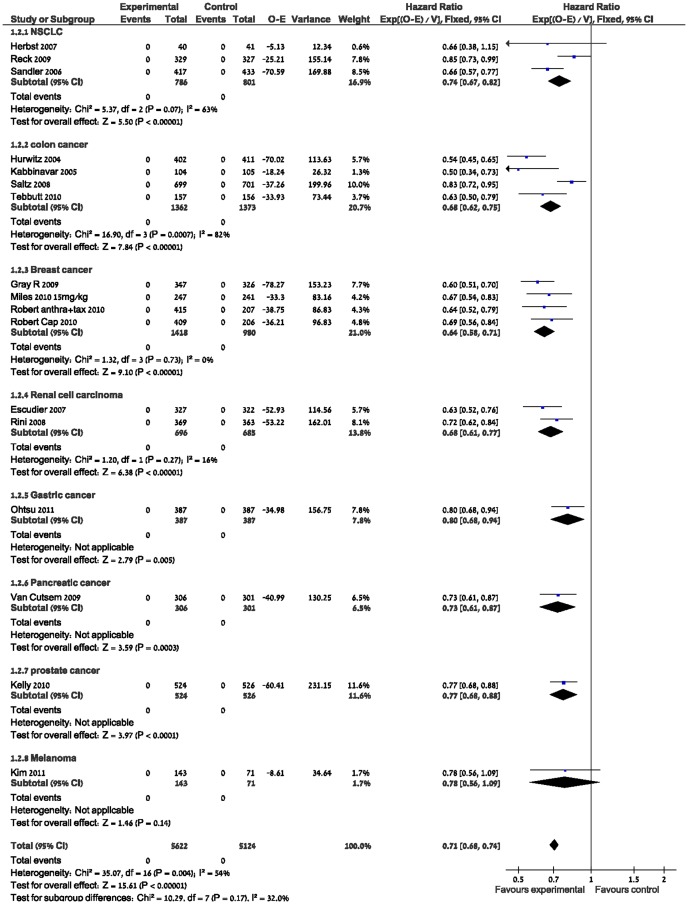
Progression free survival.

A PFS analysis of 17 of these trials including 10,746 patients (data from the CALGB pancreatic cancer trial on PFS was not published) showed a significant PFS benefit but with high heterogeneity, HR 0.71 (95% CI 0.68–0.74, p<0.00001 I^2^ = 54%). (Figures 2,3).

**Figure 4:**
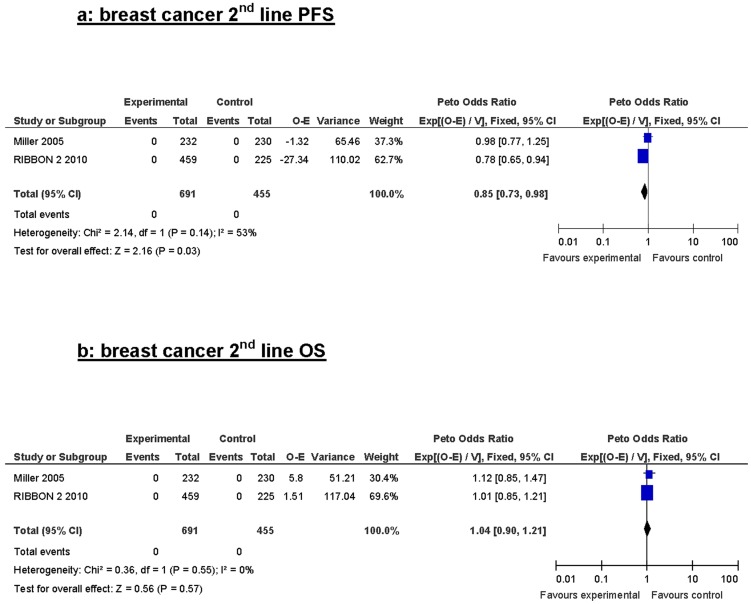
Progression free survival and overall survival in 2nd line metastatic breast cancer.

Second line treatment in breast cancer (figures 4a,4b) showed a borderline PFS advantage but no OS advantage.

### Adverse events

A meta- analysis of adverse events (AE) (table 2) showed a statistically significant increase in fatal adverse events (FAEs) in the Bevacizumab treatment arms RR 1.48 (95% CI 1.11–1.98). A separate analysis of the lung cancer trials including mainly adenocarcinoma, showed an increased risk of fatal pulmonary hemorrhage with a RR of 5.65 (95% CI 1.26–25.26).

**Table 2:** Toxicity analysis.

Event	RR	95% CI	P	I2(%)
**Adverse event >G2**	1.2	(1.15–1.24)	<0.00001	90
**Proteinuria**	7.08	(4.54–11.04)	<0.00001	0
**HTN**	4.96	(3.82–6.44)	<0.00001	0
**Hemorrhage**	1.34	(1.02–1.76)	0.28	16
**Venous thromboembolic event**	1.07	(0.9–1.27)	0.1	34
**Arterial ischemic event**	1.32	(0.98–1.78)	0.13	30
**GI perforation**	2.3	(1.34–3.95)	0.66	0
**Fatal events**	1.48	(1.11–1.98)	0.02	52
**Fatal pulmonary hemorrhage (lung studies)**	**5.65**	**(1.26–25.26)**	**0.02**	**0**

This analysis also showed an increased risk for adverse events G3–4 with Bevacizumab treatment compared with chemotherapy alone. There were 1981 events in the Bevacizumab group compared to 1619 events in the control group, RR 1.2 (95% CI 1.15–1.24). The main AEs were: hypertension (HTN) RR 4.96 (95% CI 3.82–6.44), proteinuria RR 7.08 (95% CI 4.54–11.04), hemorrhage RR-1.34 (95%CI 1.02–1.76) and GI perforation RR 2.3 (95% CI 1.34–3.95). There was no significant risk of venous thromboembolic events. Regarding arterial thromboembolic events there was no increased risk in the combined analysis but there was an increased risk in breast cancer patients RR 5.97 (95% CI 1.07–33.22) and in RCC patients RR 6.55 (95%CI 1.5–28.59).

## Discussion

The present systematic review and meta-analysis indicates that the addition of Bevacizumab to chemotherapy in patients with metastatic solid tumors resulted in a statistically significant improvement of OS and PFS. The OS benefit was significant and homogenous, with a HR 0.89 (95%CI; 0.84–0.93 P<0.00001, I^2^-4%) The effect on OS was similar for all malignancies, except for breast cancer.

The PFS benefit was significant with a HR 0.71 (95%CI 0.68–0.74 P<0.00001 I2-54%) but with high heterogeneity attributed to the NSCLC, colon and RCC trials.

There may be several potential explanations for the heterogeneity in PFS.

First, cessation of Bevacizumab treatment may lead to a rebound effect of angiogenesis, [5]. This effect might explain the different results and heterogeneity of the breast cancer trials. The E2100 breast cancer trial continued Bevacizumab until progression as compared with the AVADO breast cancer trial that gave only nine cycles of Bevacizumab. This hypothesis may justify the continuation of Bevacizumab beyond progression [23]–[24], [28] that it is currently under evaluation (Ribbon 3).

Second is the lack of predictive markers for Bevacizumab treatment. Hypertension (HTN) is known to develop in up to 30% of patients treated with Bevacizumab. Retrospective subgroup analyses have appraised the predictive value of HTN as a biomarker for response to Bevacizumab in breast and colon cancer, and have shown correlation with response but not with survival benefit [44]. A subgroup analysis of the RCC CALGB trial demonstrated a significant median OS benefit in patients with HTN≥grade 2 (according to the CTCAE 3) of 41.2 months versus 16.2 months in patients without HTN P<0.001 [44]–[46]. This putative correlation has not been evaluated in the large colon cancer studies. Other predictive parameters such as VEGF levels, vascular density or VEGF polymorphisms and molecular markers could also serve as markers of response to Bevacizumab. In the E2100 Breast cancer trial, VEGF polymorphisms (VEGF-2578 AA and VEGF-1154 AA) genotypes were associated with a superior median OS in the Bevacizumab arm compared with other genotypes (37 months and 46 months respectively compared to 25 months in the control arm) [47].

Third, the difference in the results may lie in the chemotherapy agents combined with Bevacizumab: It has been postulated that Paclitaxel given on a frequent basis also exhibits antiangiogenic and pro-apoptotic effects (partially by down-regulation of VEGF), thereby enhancing efficacy. [48]. The different result of the E2100 and the AVADO trials (breast cancer) may also be a result of the Taxane protocol used – weekly Paclitaxel as opposed to three weekly Docetaxel, an effective but toxic regimen.

In the lung cancer trials Bevacizumab again demonstrated superior OS when combined with a taxane based regimen as opposed to a gemcitabine based regimen [17]–[18].

In colon cancer trials the heterogeneity could also be explained by the different chemotherapy regimens. In Hurwitz et al; [7] the chemotherapy regimen used was IFL, known to be a toxic regimen and now rarely used whereas Saltz et al; [8] added Bevacizumab to FOLFOX.

Regarding colon cancer our results are supported by other meta-analyses evaluating the addition of Bevacizumab to chemotherapy in the metastatic setting [14], [49]–[51]

These should be interpreted in the light of the disease specific survival of the different maligancies. Therefore 3 months of survival benefit in metastatic colon cancer where the expected OS is more than 20 months differs from a three months benefit in a patient with metastatic lung or pancreatic cancer in which the median survival is less than a year [52].

The role of PFS as a surrogate for overall survival has been greatly debated in metastatic cancer. The multiple effective treatment options may explain the lack of OS advantage. Broglio et al assumed that OS represented the sum of PFS and survival post progression (SPP). He assumed that SPP contributes more to OS than PFS, therefore when SPP is 2 months there is a greater than 90% chance of detecting a statistically significant OS benefit but when SPP is 24 months there is a less than 20% chance of detecting a statistically significant OS benefit. Accordingly, breast cancer serves as a protoype for a long SPP and therefore no OS benefit was seen in these studies as compared with NSCLC with a short SPP [53].

Another important issue is that when improvements in OS are observed, their impact should be assessed considering the effect of treatment on the quality of life of the patient. Our toxicity analysis (table 2) shows an increase in adverse events >G2 RR-1.26, and fatal adverse events RR-1.47 with and increased risk for fatal pulmonary hemorrhage in the lung cancer trials with a RR-5.65. Our analysis also suggested that the most significant risk of FAEs was in patients with prostate and lung cancer, as reported by others [54]. Our analysis showed no increased risk of venous thromboembolic events, in concordance with another recently published meta-analysis [55].

Moreover our analysis showed that the different serious adverse events seemed to be more prevalent in certain tumors compared to others: G3–4 adverse events were more common with NSCLC, colon and gastric cancer. HTN was significant in all cancer types but was most significant in breast cancer with a RR-17.63, proteinuria was also significant in all types but not in gastric and colon cancer, hemorrhage was significant only in RCC, and GI perforation was significant only in colon cancer RR3.99 (95%CI1.34–11.85 p = 0.01). There was an increased risk of arterial thromboembolic events in breast cancer RR-5.97 (95% CI 1.07–33.22), and in RCC RR 6.55 (95%CI 1.5–28.59).

The calculated number needed to harm upon the incidence of fatal events is 117, i.e., for one fatal event in the bevacizumab arm 117 patients would need to be treated (RR 1.4).

The results of our analysis of Bevacizumab as second line therapy concurrently with chemotherapy in breast cancer patients showed the same trend of improved PFS and lack OS benefit, a fact that may support the use of Bevacizumab in advanced lines and could be more cost effective.

### Limitations

Limitations of our analysis should be acknowledged. The dissimilarity in the chemotherapy regimens, dosing and schedules between the various studies confound the analysis. The heterogeneous length of treatment and follow up contribute to the asymmetry as well. Also, the small number of included trials for each disease makes the outcomes more prone to be influenced by a potential publication bias. We attempted to avoid such bias by searching and including conference proceedings, databases of ongoing trials and unpublished data. The biological rationale to combine all studies in order to assess Bevacizumab universal effect might offset this limitation.

### Implications for clinical practice

This analysis showed an overall survival benefit. It reinforces the use of Bevacizumab in colon and lung cancer.

Regarding breast cancer, the FDA recently revoked its approval of the use of Bevacizumab. Our data supports this decision at this time. Promising data supporting the use of Bevacizumab in the neoadjuvant treatment of locally advanced breast cancer [56]–[57] emphasizes the need for further studies in the search of predictive markers.

Analysis of databases should be attempted in order to estimate the effect of Bevacizumab on non trial populations, as was recently published by Zhu et al [59].

### Implications for research

Many questions still remain regarding the effect of Bevacizumab as maintenance therapy compared with Bevacizumab at disease progression and the optimal schedule and type of chemotherapy in each disease. Also, Bevacizumab is a nonspecific agent without any well defined predictive markers.

Data supporting the use of Bevacizumab as neoadjuvant therapy in breast cancer patients have been published recently [57]–[58] and therefore further studies should be considered in the search for predictive markers.

Analysis of the trials already published in search of predictive markers through a patient based metaanalysis could help define possible predictive markers for future validation studies.

Finally, randomized controlled trials should have longer follow-up to appraise the long-term toxicity of Bevacizumab.

### Conclusion

In conclusion, our results suggest that adding Bevacizumab to chemotherapy results in a small but significant effect on OS and a significant PFS advantage in the advanced solid tumors included in this analysis.

## Supporting Information

Appendix S1 **Search phrase.**
(DOC)
