# Correction: The Impact of Bevacizumab (Avastin) on Survival in Metastatic Solid Tumors - A Meta-Analysis and Systematic Review

**DOI:** 10.1371/annotation/e3301fb2-ae1d-471a-aaf7-f38b4c989aff

**Published:** 2013-07-03

**Authors:** Limor Amit, Irit Ben-Aharon, Liat Vidal, Leonard Leibovici, Salomon Stemmer

The name of the fifth author was given incompletely. The correct name is: Salomon M Stemmer. 

The correct citation is: Amit L, Ben-Aharon I, Vidal L, Leibovici L, Stemmer SM (2013) The Impact of Bevacizumab (Avastin) on Survival in Metastatic Solid Tumors - A Meta-Analysis and Systematic Review. PLoS ONE 8(1): e51780. doi:10.1371/journal.pone.0051780. The correct abbreviation in Contributions is: SMS. 

