# Supplementary material for: The Impact of Bevacizumab (Avastin) on Survival in Metastatic Solid Tumors - A Meta-Analysis and Systematic Review
Source: PLoS One. 2013 Jan 22;8(1):e51780. doi: 10.1371/journal.pone.0051780 (PMC3551962; doi:10.1371/journal.pone.0051780)
Supplement: Appendix S1 — Search phrase. (DOC) [file pone.0051780.s001.doc]

Appendix 1: Search phrase((avastine OR bevacizumab) AND (neoplasm OR carcinoma OR malignan* OR tumor)))) AND (randomized controlled trial [pt]OR controlled clinical trial [pt]OR randomized controlled trial [mh]OR double-blind method [mh] OR single-blind method [mh] OR clinical trial [pt] OR clinical trials [mh] OR (“clinical trial”) [tw] OR singl* [tw] OR doubl* [tw] OR trebl* [tw] OR tripl* [tw] AND (mask* [tw] OR blind* [tw]))) OR (placebos [mh] OR placebo* [tw] or random* [tw] PR research design [mh:noexp] OR comparative study [mh] OR evaluation studies [mh] OR follow up studies [mh] OR prospective studies [mh] OR control* [tw] OR prospective* [tw] OR volunteer* [tw] NOT (animals [mh] NOT humans [mh]).
